# A Comparison in Prevalence of *Helicobacter pylori* in the Gingival Crevicular Fluid from Subjects with Periodontitis and Healthy Individuals using Polymerase Chain Reaction

**DOI:** 10.5681/joddd.2013.038

**Published:** 2013-12-18

**Authors:** Mohammad Reza Salehi, Mohammad Shah Aboei, Narges Naghsh, Samira Hajisadeghi, Ebrahim Ajami

**Affiliations:** ^1^Assistant Professor, Department of Oral & Maxillofacial Medicine & Torabinejad Dental Research Center, School of Dentistry, Isfahan University of Medical Sciences, Isfahan, Iran; ^2^Associate Professor, Department of Periodontics, School of Dentistry & Torabinejad Dental Research Center, Isfahan University of Medical Sciences, Isfahan, Iran; ^3^Assistant Professor, Department of Periodontics, School of Dentistry, Isfahan University of Medical Sciences, Isfahan, Iran; Assistant Professor, Department of Oral & Maxillofacial Medicine, School of Dentistry, Qom University of Medical Sciences, Qom, Iran ^4^; ^5^Dentist, Private Practice, Isfahan, Iran

**Keywords:** Helicobacter pylori, gingival crevicular fluid, periodontal disease, PCR

## Abstract

***Background and aims.*** The high prevalence of *Helicobacter pylori* among the microorganisms isolated from the oral environment brings up the question of whether oral cavity acts as a reservoir for this bacterium. The aim of the present study was to determine and compare the prevalence of *H. pylori *in gingival crevicular fluid (GCF) of patients with chronic periodontitis (CP) as an infectious disease and healthy subjects using polymerase chain reaction (PCR).

***Materials and methods.*** Periodontal examination was performed for all participants. Two sterilized paper points were inserted to the maximum depth of the periodontal pockets of selected teeth. The presence of *H. pylori* was determined by PCR. In the CP group, the severity of disease was defined as moderate or severe. Further, the frequency of Helicobacter pylori in GCF of each category, and the association between the presence of Helicobacter pylori in GCF and periodontitis were determined.

***Results. ***There was no statistically significant association between CP and the presence of *H. pylori* in the GCF (P = 0.62), there was no significant correlation between the presence of *H. pylori* in the GCF and gender of the subjects (P = 0.28 in CP group and P = 0.25 in control group), and there was no significant correlation between the presence of *H. pylori* in the GCF and severity of periodontitis (P = 0.20).

***Conclusion.*** Oral cavity acts as a reservoir for *H. pylori*; however, the results do not show that *H. pylori *is involved in periodontal disease.

## Introduction

Studies suggest that the periodontal diseases, particularly chronic periodontitis (CP) are infectious diseases caused by particular pathogens or various microorganisms in the periodontal tissues. In this regard, certain microorganisms have been identified as etiologic agents of periodontitis –some for sure and some probably- and researchers believe that probably other microorganisms can increase the risk of periodontitis. Isolation of diverse and numerous microorganisms in periodontal pockets or in dental plaque highlight the importance of the above mentioned point.^1^

*Helicobacter pylori* is a fastidious, microaerophilic, gram-negative spiral shaped bacterium originally classified as *Campylobacter pyloridis*. Nearly 10% of individuals are affected by gastritis and/or gastric ulcer during their lifetime, and more than 50% of the world’s population carries this infection.^2^ The oral cavity has been mentioned as the primary extra gastric region with great potential to harbor *H. pylori *and cause re-infection. It has been the focus of many studies,^3,4^ and it seems that dental plaque has a more important role for harboring* H. pylori*.^5,6^

Periodontitis is a common oral disease and a high amount of plaque is detected in periodontal patients. The high prevalence of *H. pylori* among the microorganisms isolated from the oral environment, saliva and dental plaque, brings into mind the question as to whether this bacterium is considered as a part of the normal oral flora or not. The microorganism’s association with many diseases has been documented in the recent years. Also, the fact that the microorganism is not essentially acidophilic and can also be present in other parts of the body including oral cavity, increases the possibility of the virulence of the microorganism in other environments in addition to the stomach and intestines.^7^Researchers have emphasized the relationship of this microorganism with gastritis, dyspepsia, gastric lymphoma and cancer, gastric and duodenal ulcers and pancreatic cancer. Epidemiological studies show that *H. pylori* is found commonly everywhere and about half of humans worldwide are infected with this bacterium.^2^

Certain microorganisms are involved in periodontitis and it is associated with some systemic diseases.^8^ The economic, social and psychological costs of both periodontitis and *H. pylori* infections for the individual and the society are remarkable, and the prevalence of *H. pylori* in the stomach and oral cavity is high. Therefore, probable relationship between *H. pylori* and periodontitis should be assessed, and identifying any association could be regarded as a way to prevent, control and manage both the *H. pylori* infection and the periodontitis. Based on this scientific and practical necessity, it would be interesting to evaluate the prevalence of* H. pylori* in chronic periodontitis. This research sought to find out the prevalence of *H. pylori* in gingival crevicular fluid (GCF) regardless of the patient’s stomach situation. Another purpose was to compare the prevalence of *H. pylori* in GCF of periodontal patients with that of a control group detected by polymerase chain reaction (PCR).

## Materials and Methods

### Subjects

The present research is a case-control study and the study protocol was approved by the ethical committee and the research vice chancellor of Isfahan University of Medical Sciences. Our research participants were volunteer patients who referred to the Department of Periodontology, School of Dentistry, Isfahan University of Medical Sciences, Isfahan, Iran. The patients were selected by convenience sampling, and the questionnaire was completed for each patient using a specific code.

Exclusion criteria were: age of under 20 years, having ≤ 10 natural teeth, history of gastric symptoms and use of inhibitors of proton pomp, H2 blockers and bismuth derivates, use of antimicrobial agents within 6 months prior to the study, previous upper digestive hemorrhage and gastric cancer, presence of underlying systemic diseases such as diabetes mellitus, pregnant women, HIV-positive patients, smokers and other systemic conditions that could affect the periodontal status, edentulous patients or patients with aggressive periodontitis, and history of previous scaling and root planning or periodontal therapy in the last 6 months. Periodontal examination was performed for all participants and measurements were taken at six sites per tooth (mesio-buccal, mesio-lingual, disto-buccal, disto-lingual, mid-buccal and mid-lingual), using a Williams periodontal probe (Williams periodontal probe, Hu-Friedy, Chicago, IL.). Patients presenting 3 mm clinical attachment loss (CAL) within at least four teeth and exhibiting more than 10% of sites with bleeding on probing (BOP) were diagnosed as periodontitis (N = 50), and patients presenting less than 3 mm CAL within at least four teeth with or without bleeding were allocated to the periodontally healthy (N = 50).^9^

### GCF Sampling

Previously selected teeth (4 teeth in each quadrant: central incisor-canine-second premolar and first molar) were isolated with sterile cotton rolls, and the supragingival plaque was removed with sterile cotton pellets. Two sterilized paper points (#30) were carefully inserted into the maximum depth of the periodontal pocket and held in position for 20 s.

### Extraction of DNA

DNA was extracted from GCF of *H. pylori* by the method described by Willis et al.^10^with some of our own minor modifications. We used a commercial kit (Kiagene, USA). Briefly, GCF samples were placed into sterile tubes containing 0.5 ml of trypticase soy broth, and then vortex was mixed for 30 seconds and boiled for 5 minutes.

### PCR Amplification

The pair of primers were JW22 (5-CGTTAGCTGCATTACTGGAGA-3) and JW23 (5-GAGCGCGTAGGCGGGATAGTC-3). PCR Amplification was performed in a reaction volume of 50 μl consisting of 5 μl of plaque lysate or 1 μl of *H. pylori *genomic DNA and either 45 or 49 μl of reaction mixture containing 1x PCR buffer, 1 unit of Taq DNA polymerase, 0.2 μM of each deoxinucleotide triphosphates and each primer at a concentration of 0.2 μM. After an initial denaturation step of 95 C for 5 minutes, there were 40 cycles of denaturation steps at 94 C for 1 minute, annealing at 60 C for 1 minute and extention at 72 C for 1 minute, followed by a final extension step at 72 C for 10 minutes. The presence of bacterial DNA was determined by an amplicon of 295bp in size visualized on a 2% agarose gel.

### Statistical Analysis

Data were entered into SPSS software. The Chi-square test was employed. In the CP group, the severity of disease was defined as moderate (3 to 4 mm of CAL) or severe (CAL≥5 mm).^11^ Also, the frequency of *H. pylori *in GCF in each category, and the association between the presence of *H. pylori* in GCF and periodontitis were determined. The significance level was set at p <0.05.

## Results

Of 100 studied population 42 subjects were males and 58 were females and mean age of them was 35.3 ± 10.6. Demographic characteristics of the study population have been briefly mentioned in Table 1. The mean of age between groups was statistically significant but composition of sex in case and control groups was similar.

**Table 1:** Demographic Features of Periodontally Health Subjects and Subjects with CP

Characteristics	Groups	P-value
	Chronic priodontitis (case) N=50	Periodontally health (control) N=50	
Age (year)	42.6 ± 9.4	28.5 ± 9.5	<0.0001^*^
Sex (female / male)	28/22	30/20	0.84†
Severity of periodontitis (moderate / severe)	34/16	—	—
Data are presented as mean ± SD, count and number (percent).
P-values calculated by *Independent sample t-test, and †Chi-square.

*H. pylori* was detected in the GCF of 21 patients of all studied groups (21%). 9 out of 21 patients (18%) had periodontaitis, while the other 12 patients (24%) were periodontally normal ( Table 2). There was no statistically significant association between periodontal disease and the presence of* H. pylori* in the GCF (P = 0.62). Also, the subjects were classified according to gender and severity of periodontaitis (moderate and severe) and the presence of *H. pylori* was evaluated in each group. Chi-square test results showed that there is no significant correlation between the presence of *H. pylori* in the GCF and gender of the subjects (P=0.28 in CP group and P=0.25 in control group). Among the samples with periodontal disease, 34 of them (68%) had moderate periodontitis and 16 samples (32%) had severe periodontitis. *H. pylori* was detected more often in the GCF from subjects with severe periodontitis (31%) compared to moderate periodontitis (11%). But Chi-square test results showed that there is no significant correlation between the presence of *H. pylori* in the GCF and severity of periodontitis (P= 0.20).

**Table 2:** Distribution of samples according to RCR results based on study groups

PCR result	Groups	P-value
	Chronic periodontitis (case) N=50	Periodontally health (control) N=50	
PCR+	9(18)	12(38)	0.62
PCR−	41(82)	38(76)	
Data are presented as Number (percent).
P-values calculated by Chi-square.

## Discussion

Some studiessuggested that the oral cavity may be a reservoir for *H. pylori* and the presence of *H. pylori* in oral cavity can act as a possible source of recontamination of the treated patient; it can transfer from one person to another and we should pay attention to detect the bacteria in the oral cavity. But other researchers believe that this bacterium has only a transient presence in the oral environment.^4,13^ In addition, it is possible that the prevalence of H. pylori in the oral cavity is affected by the presence of oral infection such as periodontitis. Another question arises as to whether *H. pylori* causes periodontitis and vice versa. Since data correlating periodontal disease and *H. pylori* colonization are limited,^3,4^ we evaluated the prevalence of* H. pylori* in the oral cavity (especially GCF) regardless of the status of their stomach and the potential association between the presence of *H. pylori* in gingival GCF and chronic periodontitis. Our results support the data in the literature showing the presence of *H. pylori* in the oral cavity, particularly in the GCF.^3,4,14^This is because the prevalence of *H. pylori* in the total population was 21% but there was no significant difference between CP and the periodontal health group in this respect.

The survey results are in agreement with the results of Souto and Colombo’s study which suggested that the oral cavity may act as a reservoir for H. pylori, and that failure to eliminate this specie from the mouth could lead to recolonization and re-infection in the stomach. So it is better to remove the bacteria from the oral cavity to prevent relapses of the gastrointestinal (GI) infections.

Clearly, the prevalence of *H. pylori* will vary significantly between different population groups and this may account partly for the conflicting data in the literature about the presence of this organism in the oral cavity. Variations in *H. pylori* detecting methods in the oral cavity, diversity in the studied population, type and number of clinical samples, oral health status, the classification of periodontal disease and also the technical difficulties may cause different results in research.^5^ Microbiological culture is the gold standard method for detecting the presence of H. pylori, but some forms of microorganisms are difficult or impossible to culture.^2^ Some researchers use the rapid urease test for investigating the presence of H. pylori.^6^ This method is simple, but there are many bacteria in the oral cavity that produce urease enzymes, which can lead to false positive results. Some researchers used the PCR technique for investigating H. pylori.^3,6,14,15^ Although in comparison with culture and rapid urease test, PCR is non- invasive, gives the highest detection rate, and plays a significant role in the early detection of *H. pylori* infection, its high sensitivity can lead to false positive reactions as a result of sample contamination by PCR products and the specificity and sensitivity may vary among sets of primers and results obtained with detection methods based only on PCR should be interpreted with caution because other microorganisms that are phylogenetically very close to *H. pylori* are also present in the mouth.^12^ The primers used in this study (JW22 and JW23) have been tested for their specificity and sensitivity.^3,7^ In symptomatic individuals, PCR has detected *H. pylori* in saliva, whereas culture methods have very rarely isolated *H. pylori* from saliva.^16,17^ Finally, in the current study, the PCR method was used for *H. pylori* detection.

Another issue is that periodontitis and *H. pylori* infections can be influenced by different risk factors, such as age, ethnicity, gender and socioeconomic status.^18^ Associations between age or gender and the frequency of *H. pylori* showed a slightly greater prevalence in males (31%) than in females (21%), as well as in older individuals (>50 years of age; 31%) in comparison with younger individuals (18 to 30 years of age; 13.5%). On average, most of the subjects in our study had low socioeconomic level, but no significant differences were found between the two groups for these variables. In a large epidemiological study, Dye et al.^4^ found a significant association between the prevalence of *H. pylori* and advanced periodontal diseases even after adjusting for these socio-demographic factors.

Some investigators believe that the periodontal pocket is a rich environment for the colonization of H. pylori. The variety of microbes with the permanent inflammatory process can lead to appropriate sites for the establishment of the microorganism. Closely related species (i.e Campylobacter spp.) increase in frequency and number in sites with deep pockets that lack periodontal attachment and BOP.^19^ Andersen et al. reported that some species of the orange complex (Fusobacterium spp.) coaggregated with H. pylori.^20^ These species produce several metabolites, such as formate and fumarate that are used as energy sources by species of Campylobacter and Wollinella. Thus, colonization by Fusobacterium may favor the establishment of* H. pylori* in the periodontal pocket^21^ and moreover, the subgingival biofilm can provide a significant amount of urea, which can select for urease producing bacteria such as H. pylori. Antagonistic relationships also may occur within the subgingival biofilm. Okuda et al.^8^ observed that oral streptococci produce bacteriocin-like inhibitory proteins against H. pylori. Subjects with proper oral hygiene house less *H. pylori* in their mouths also due to this inhibitory activity of the early colonizers of dental biofilm such as oral streptococci. Clinically, the periodontal health subjects had minimal dental biofilm accumulation and gingival inflammation, which were compatible with an adequate oral hygiene.

One of our goals was to determine whether oral cavity could act as a reservoir for *H. pylori* or not, regardless of their stomach infection. In contrast to most of the reported studies, we evaluated subjects who reported no history of gastric symptoms. Berroteran et al.^22^ detected *H. pylori* in the biofilm of 15% of asymptomatic subjects, whereas Martinez-Gomis et al.^7^ did not detect this bacterium in any of the 10 subjects without dyspepsia they evaluated. We found a much higher overall prevalence (20%) in our total population without dyspepsia; specifically, in 18% of CP patients and in 24% of periodontal health patients. Conversely, Olivier et al.^13^ did not detect *H. pylori* in dental samples from subjects without periodontitis who had positive stomach biopsies. Umeda et al.^14^ reported that a subject with periodontal pockets retained *H. pylori* in the oral cavity, even after eradication of the bacterium from the stomach. Gebara et al. showed that after triple therapy, 60% of patients with periodontaitis remained positive for *H. pylori* DNA in their oral cavity, whereas only 10% were positive in their stomach.^23^ These controversial findings may suggest that the frequency of *H. pylori *in the oral cavity is related more to the presence of periodontal disease than to the existence of gastric infection. Moreover, eradication of this specie from the subgingival biofilm of subjects with periodontal disease may be limited.

The prevalence of *H. pylori *and chronic periodontitis are common among the general population,^24^ but there are contradicting data about the correlation between them. These controversies indicate that the potential relationship between them is more complex.

According to our data, there are no significant differences in the presence of* H. pylori* between healthy individuals and periodontitis patients, so we cannot say that *H. pylori* could certainly be involved in causing periodontal disease. The survey results are in agreement with the results of the data showing the presence of *H. pylori* in the GCF of patients with CP and poor oral hygiene.^3,4,14,25^ Riggio and Lennon observed *H. pylori* in the subgingival biofilm of 38% of the periodontitis patients,^25^ whereas Dye et al^4^ and Gebara et al^3^ detected the bacterium in 41% and 26.6% of these subjects, respectively.

The advantageous distinction of our study in comparison with previous studies was the use of the control group. In our study, *H. pylori* was detected in approximately 20% of periodontitis patients, whereas its prevalence was not more significant than periodontal health (control). In previous studies, there had been less usage of control groups. In some research, *H. pylori* is mentioned as the normal flora of the mouth and perhaps is not one of the etiologic or pathologic factors of periodontal diseases and is like many of the Non-pathogenic microbes of the mouth. To investigate this discrepancy, we used one control group and the presence of *H. pylori* was detected in the control group similarly and since there is no plaque and calculus in the control group, we sampled from the GCF. Finally our investigation showed that there are no significant differences in the presence of* H. pylori *between healthy individuals and periodontitis patients.

## Conclusion

*H. pylori *was detected frequently in the oral microbiota of subjects, suggesting that oral cavity acts as a permanent reservoir for this bacterium. But there are no significant differences in the presence of *H. pylori* between healthy individuals and periodontitis patients, and therefore, no claim can be made on certain involvement of *H. pylori *in causing periodontal disease.
